# No change in interictal C-reactive protein levels in individuals with episodic and chronic migraine: A case-control study and literature review

**DOI:** 10.3389/fneur.2022.1021065

**Published:** 2022-10-12

**Authors:** Chae Gyu Park, Sue Hyun Lee, Min Kyung Chu

**Affiliations:** ^1^Heart-Immune-Brain Network Research Center, Department of Life Science, Ewha Womans University, Seoul, Republic of Korea; ^2^Laboratory of Immunology, Severance Biomedical Science Institute, Yonsei University College of Medicine, Seoul, Republic of Korea; ^3^Therapeutic Antibody Research Center, Genuv Inc., Seoul, Republic of Korea; ^4^Department of Neurology, Yonsei University College of Medicine, Seoul, Republic of Korea

**Keywords:** biomarker, C-reactive protein, inflammation, migraine, headache

## Abstract

**Objectives:**

The levels of some migraine biomarkers differ between episodic migraine (EM) and chronic migraine (CM), but information on C-reactive protein (CRP) levels in EM and CM is conflicting. Thus, this study aimed to evaluate CRP levels in participants with EM and CM in comparison to those in healthy controls.

**Methods:**

Plasma CRP levels were evaluated by high-sensitivity CRP tests in female participants with EM (*n* = 174) and CM (*n* = 191) and healthy controls (*n* = 50).

**Results:**

The results showed no significant difference in CRP levels among the EM, CM, and control groups (median and interquartile range, 0.40 [0.15–0.70] mg/L vs. 0.40 [0.15–1.00] mg/L vs. 0.15 [0.15–0.90] mg/L, *p* = 0.991). The ratio of individuals with elevated CRP levels (>3.0 mg/L) did not significantly differ among the EM, CM, and control groups (3.4% [6/174] vs. 2.1% [4/191] vs. 0.0% [0/50], *p* = 0.876). Multivariable regression analyses revealed that CRP levels were not significantly associated with headache frequency per month (β = −0.076, *p* = 0.238), the severity of anxiety (Generalized Anxiety Disorder-7 score, β = 0.143, *p* = 0.886), and depression (Patient Health Questionnaire-9 score, β = 0.143, *p* = 0.886). Further, CRP levels did not significantly differ according to clinical characteristics, fibromyalgia, medication overuse, preventive treatment, and classes of preventive treatment medications. Among participants with a body mass index ≥25 kg/m^2^, the CRP levels in EM (*n* = 41) and CM (*n* = 17) were numerically higher than those in the control (*n* = 6) (1.30 [0.28–4.25] mg/L vs. 1.10 [0.50–3.15] mg/L vs. 0.40 [0.15–0.83] mg/L, *p* = 0.249) but did not reach statistical significance.

**Conclusions:**

The interictal CRP level is not likely to be a biomarker for EM or CM.

## Introduction

C-reactive protein (CRP) is an acute-phase protein whose circulating level rises in response to acute and chronic inflammation (1). It originates from the liver following the secretion of interleukin-6 (2). As an inflammatory biomarker, CRP is non-specific (3). Recent studies have revealed that increased CRP levels may reflect vascular inflammation. Inflammation plays an essential role in the pathogenesis of vascular diseases (3, 4). CRP levels are also a powerful predictor of cardiovascular disease and metabolic syndrome (5, 6). In addition, a significant relationship between migraine and cardiovascular disorders has been described (7, 8). Further, high CRP levels have been reported in patients with migraine (9–15). Increased CRP levels are a marker of systemic inflammation. Since systemic inflammation can contribute to an additional risk of cardiovascular disorder, increased CRP levels in migraine cases have been used to explain the relationship between migraine and cardiovascular diseases (11, 16, 17). However, several studies have reported that CRP levels between individuals with migraine and controls are unrelated (18–25).

Migraine is classified as episodic migraine (EM) and chronic migraine (CM) (26), and there are prevalence, comorbidities, and treatment response differences between the two (27). Patients with CM have a higher tendency to develop central sensitization and pain transmission than patients with EM (28). Previous studies on migraine biomarkers also revealed discrepancies in some biomarkers between EM and CM (29, 30). Nevertheless, information on CRP levels in cases of EM and CM is currently limited. Thus, this study aimed to evaluate plasma CRP levels in patients with EM and CM compared to controls. Additionally, the relationship of CRP levels with clinical characteristics and comorbidities was also investigated. We hypothesized that the CRP levels in participants with EM and CM were higher than in controls.

## Materials and methods

### Study design and participants

This was a cross-sectional case-control study of consecutive female patients with EM or CM who visited an outpatient clinic of the Department of Neurology, Severance Hospital, Yonsei University College of Medicine, from June 2019 to October 2020. The inclusion criteria were defined as follows: (1) age, 19–65 years; (2) migraine diagnosis according to the third edition of the International Classification of Headache Disorders (ICHD-3) for EM (code 1.1 or 1.2) or CM (code 1.3) (26); (3) >48 h had passed since the last migraine attack for participants with EM and CM; (4) types and dosage of any preventive drugs were stable for ≥1 month; and (5) full cooperation of the study protocol. The exclusion criteria were defined as follows: (1) secondary headache with the exception of medication-overuse headache (MOH) defined in the ICHD-3 (26), (2) chronic pain with the exception of fibromyalgia (FM) based on the 2016 American College of Rheumatology criteria (31), and (3) ongoing medical and psychiatric treatment. For healthy controls, individuals were considered qualified if they did not have any form of headache throughout the previous year and did not report a migraine attack during their lifetime. Healthy controls having any inflammatory or systemic diseases were not included. Healthy controls were enrolled by advertisement.

### Sample size estimation

The sample size was calculated based on a past study on the interictal level of CRP in patients with migraine (13). The proportion of migraines to controls was set at 1:1. Using a 5% significance level and 80% power, the sample sizes of the migraine and control groups were calculated to be 45 and 45, respectively. Our target was to enroll 150 patients per migraine group to evaluate the relationship between CRP levels and various clinical characteristics, in addition to the difference in CRP between healthy controls and the migraine groups. Moreover, in the case of healthy controls, our target was to enroll 50 participants.

### Plasma collection and CRP measurement

Blood samples were collected from the right antecubital vein into tubes with ethylenediamine tetraacetic acid. After centrifugation at 3,500 rpm for 10 min at 4°C, plasma was collected. The CRP level was measured using the high-sensitivity CRP (hs-CRP) test on a Roche Cobas C701 module (Roche Diagnostics International AG, Zug, Switzerland). As the reagent, Cobas C-Reactive Protein Gen.3 was used.

### Assessment of anxiety, depression, FM, and medication overuse

Anxiety and depression were assessed as they are both prevalent in individuals with migraine and have a close relationship to the severity of migraine (32). The severity of anxiety was assessed using the Generalized Anxiety Disorder-7 (GAD-7), with anxiety defined as a GAD-7 score ≥8 (33, 34). Meanwhile, the severity of depression was assessed using the Patient Health Questionnaire-9 (PHQ-9), and depression was defined as a PHQ-9 score ≥10 (31). FM was diagnosed by the 2016 American College of Rheumatology criteria (31). Medication overuse (MO) was classified by the MOH criteria (code 8.2) as follows: repetitive intake of triptans, ergotamine, combination analgesics, and opioids for ≥10 days/month or repetitive intake of non-opioid analgesics for ≥15 days/month for >3 months. If a participant used multiple drug classes, the MOH criteria attributed to multiple drug classes were used instead of the individually overused criteria (code 8.2.6) (26). Headache day frequency (≥15 days/month) was not included as a criterion for the diagnosis of MO.

### Statistical analysis

Binary and ordinal scales are presented as numbers and percentages. When comparing the sizes between sample groups, the Kolmogorov test was used when a normality test of continuous variables was required. Normally distributed continuous variables were compared using an independent *t*-test or analysis of variance (ANOVA), and values are presented as the means ± standard deviations. Meanwhile, non-normally distributed variables were compared using the Mann–Whitney *U*-test or Kruskal–Wallis test, and values are expressed as the medians (interquartile range [IQR]). The chi-square test was used to compare categorical variables. If the residuals from the model satisfied parametric assumptions, we performed an ANOVA or analysis of covariance (ANCOVA). Although the model did not help parametric assumptions, ANCOVA was performed because it was robust when the assumptions were not satisfied (35). Similar analyses and adjustments were used to evaluate the association of CRP with clinical characteristics, comorbidities, and preventive medication classes.

The proportion of elevated CRP levels was also compared using a multivariable regression analysis with age and body mass index (BMI) adjustments. *Post-hoc* analyses were performed using Bonferroni's method. For *post-hoc* analyses comparing CRP levels among the groups, a significance level of *p* < 0.017 was applied (0.050/3). All statistical analyses were performed using IBM SPSS software version 25 for Windows (IBM Corp., Armonk, NY, USA). A two-tailed *p* < 0.05 was considered statistically significant.

This is the primary analysis of our collected data. We preplanned the analysis processes before the data collection. There were no missing data in our study.

## Results

### Clinical characteristics and demographics of participant

A total of 415 female participants were enrolled; 50, 174, and 191 belonged to the control, EM, and CM groups, respectively. The clinical characteristics and demographics are summarized in Table 1. Age and BMI discrepancies among the three groups were statistically significant. *Post-hoc* analyses showed that age (*p* = 0.013) was significantly lower while BMI (*p* = 0.006) was significantly higher in the EM group than in the CM group. However, neither age nor BMI significantly differed between the EM and control groups or between the CM and control groups. Unilateral pain and aura were more prevalent in the EM group than in the CM group.

**Table 1:** Clinicodemographic participant characteristics by group.

	**Episodic migraine group, n = 174**	**Chronic migraine group, n = 191**	**Control group, n = 50**	***P* value**
Age (years)	42.00 (30.00–49.25)	44.00 (35.00–53.00)	45.00 (31.75–53.25)	0.044* Control vs. EM = 0.299 Control vs. CM = 0.481 EM vs. CM = 0.013
Body mass index (kg/m^2^)	21.16 (19.57–23.27)	22.15 (19.94–24.46)	21.96 (19.90–23.85)	0.043* Control vs. EM = 0.174 Control vs. CM = 0.710 EM vs. CM = 0.015
Headache frequency per month	4.00 (3.00–8.00)	30.00 (19.00–30.00)		< 0.001^†^
Headache intensity				0.104^†^
Mild	3 (0.0)	1 (1.1)		
Moderate	29 (16.7)	20 (10.5)		
Severe	137 (78.7)	166 (86.9)		
Unilateral pain	106 (60.9)	69 (36.1)		< 0.001^†^
Pulsating quality	168 (96.6)	182 (95.3)		0.544^†^
Aggravation by movement	125 (71.8)	167 (87.4)		< 0.001^†^
Nausea	162 (93.1)	181 (94.8)		0.505^†^
Vomiting	60 (34.5)	78 (40.8)		0.211^†^
Photophobia	75 (43.1)	105 (55.0)		0.023^†^
Phonophobia	81 (46.6)	120 (62.8)		0.002^†^
Anxiety (GAD-7 score ≥ 8)	30 (17.2)	96 (50.3)		< 0.001^†^
Depression (PHQ-9 score ≥ 10)	32 (18.4)	68 (35.6)		< 0.001^†^
Fibromyalgia	16 (9.2)	69 (36.1)		< 0.001^†^
Preventive medications	44 (25.3)	67 (35.1)		0.042^†^
Medication overuse	3 (1.7)	39 (20.4)		< 0.001^†^
Migraine with aura	18 (10.3)	1 (0.5)		< 0.001^†^

*Comparison among all three groups.

^†^Comparison between participants with episodic and chronic migraine.

Values are given as the medians and interquartile ranges or *n* (%).

CM, chronic migraine; EM, episodic migraine; GAD-7, Generalized Anxiety Disorder-7; PHQ-9, Patient Health Questionnaire-9.

Among the participants with migraine, 42 (11.5%) and 85 (23.3%) participants in the EM and CM groups were diagnosed with MO and FM, respectively. Anxiety and depression were diagnosed in 126 (34.5%) and 100 (27.4%) participants, respectively. None of the control group participants had anxiety, depression, or FM.

### CRP levels

**Figure 1:**
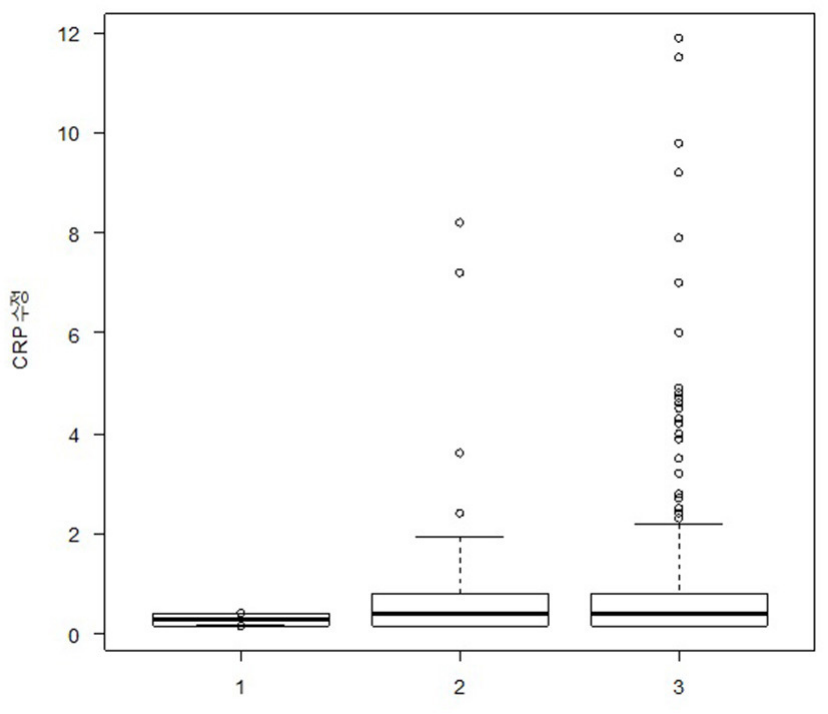
Box plot showing plasma glutamate levels in participants with episodic migraine (*n* = 174), participants with chronic migraine (*n* = 191), and healthy controls (*n* = 50). The box boundary closest to zero indicates the 25th percentile in the box plots. The black line within the box marks the median. The boundary of the box farthest from zero indicates the 75^th^ percentile. Whiskers above and below the box indicate the 10^th^ and 90^th^ percentiles, respectively.

The median (IQR) plasma CRP levels in the EM, CM, and control groups were 0.40 (0.15–0.70) mg/L, 0.40 (0.15–1.00) mg/L, and 0.15 (0.15–0.90) mg/L, respectively, with no significant differences after adjusting for age and BMI in the ANCOVA (*p* = 0.991) (Figure 1). The ratio of participants with elevated CRP (>3.0 mg/L) was also not significantly different among the EM, CM, and control groups (3.4% [6/174] vs. 2.1% [4/191] vs. 0.0% [0/50], *p* = 0.876).

Similarly, after excluding 113 (27.2%) participants with MO or FM, no significant differences in CRP levels were observed among the EM, CM, and control groups after adjusting for age and BMI in the ANCOVA (0.4 [0.15–0.78] mg/L vs. 0.40 [0.15–0.80] mg/L vs. 0.40 [0.15–0.70] mg/L, *p* = 0.722). Moreover, there were also no significant differences in the proportion of participants with high CRP levels (3.2% [5/155] vs. 3.1% [3/97] vs. 0.0% [0/50], *p* = 0.452).

Among the 66 participants who were at least overweight (i.e., BMI ≥ 25 kg/m^2^) (EM group, *n* = 41; CM group, *n* = 17; and control group, *n* = 8), the CRP levels of the EM and CM groups were numerically higher than those of the control group but did not reach statistical significance after adjusting for age and BMI (1.30 [0.28–4.25] mg/L vs. 1.10 [0.50–3.15] mg/L vs. 0.40 [0.15–0.83] mg/L, *p* = 0.249).

### Association of CRP levels with headache frequency, headache intensity, BMI, and severity of anxiety and depression

A multivariable linear regression analysis that included age, BMI, headache frequency, and the severity of anxiety (GAD-9 score) and depression (PHQ-9 score) revealed that there was a significant positive linear relationship between CRP levels and BMI (33, 36). However, no significant linear relationship was found between CRP levels and headache frequency, the severity of anxiety, or depression (Table 2). CRP levels did not vary significantly according to headache intensity (mild, 0.28 [0.15–0.28] mg/L vs. moderate, 0.40 [0.15–0.85] mg/L vs. severe, 0.40 [0.15–0.80] mg/L, *p* = 0.542).

**Table 2:** Age-adjusted multivariable regression analysis of the association of C-reactive protein levels with body mass index, headache frequency per month, and the severity of anxiety and depression.

	**Unstandardized coefficients**		**Standardized coefficients**	**t**	***P* value**
	**B**	**Standard error**	**β**		
Body mass index	0.158	0.026	0.368	5.957	< 0.001
Headache frequency per month	−0.009	0.007	−0.076	−1.183	0.238
The severity of anxiety (GAD-7)	0.003	0.020	0.014	0.143	0.886
The severity of depression (PHQ-9)	−0.004	0.023	−0.016	−0.0173	0.862

GAD-7, Generalized Anxiety Disorder-7; PHQ-9, Patient Health Questionnaire-9.

### CRP levels in compliance with clinical characteristics, migraine comorbidities, and preventive medications

The CRP levels in compliance with clinical characteristics and migraine comorbidities are summarized in Table 3. Multivariable linear regression analyses modified by age and BMI revealed that CRP did not significantly vary in compliance with clinical characteristics and comorbidities in the 365 participants with migraine.

**Table 3:** C-reactive protein levels of the 365 participants with migraine (episodic migraine or chronic migraine) by clinical characteristics and comorbidities.

	**With relevant characteristics, (mg/L), median and interquartile range**	**Without relevant characteristics, (mg/L), median and interquartile range**	*P* value*
Unilateral pain	0.40 (0.15–0.80)	0.40 (0.15–0.90)	0.927
Pulsating quality	0.40 (0.15–0.80)	0.15 (0.15–0.80)	0.434
Aggravation by movement	0.40 (0.15–0.80)	0.40 (0.15–0.80)	0.401
Severe intensity	0.40 (0.15–0.80)	0.40 (0.15–0.85)	0.095
Nausea	0.40 (0.15–0.83)	0.28 (0.15–0.63)	0.458
Vomiting	0.40 (0.15–0.75)	0.40 (0.15–0.90)	0.309
Photophobia	0.40 (0.15–0.80)	0.40 (0.15–0.80)	0.740
Phonophobia	0.40 (0.15–0.90)	0.40 (0.15–0.80)	0.707
Anxiety (GAD-7 score ≥ 8)	0.15 (0.15–1.00)	0.40 (0.15–0.80)	0.119
Depression (PHQ-9 score ≥ 10)	0.40 (0.15–1.05)	0.40 (0.15–0.80)	0.253
Fibromyalgia	0.45 (0.15–1.08)	0.40 (0.15–0.80)	0.091
Medication overuse	0.40 (0.15–0.83)	0.40 (0.15–0.80)	0.689
Migraine with aura	0.40 (0.15–0.90)	0.40 (0.15–0.80)	0.367

*p-value: from analysis of covariance between groups, with age and body mass index as covariates. GAD-7, Generalized Anxiety Disorder-7; PHQ-9, Patient Health Questionnaire-9.

Evaluation of the CRP levels according to preventive medication classes showed that 111 (30.4%) patients with EM or CM took preventive medications. No participant used anti-calcitonin gene-related peptide antibody or botulinum toxin A for migraine treatment. In a multivariable regression analysis, CRP levels did not significantly vary in compliance with preventive medication classes (Table 4).

**Table 4:** C-reactive protein levels among the 365 participants with migraine (episodic migraine or chronic migraine) by class of preventive medications.

	**Number (%) of participants using the corresponding class of preventive medications**	**Plasma levels in users of the corresponding class of preventive medications (mg/L)**	**Plasma levels in non-users of the corresponding class of preventive medications (mg /L)**	*P* value*
All preventive medications	111 (30.4)	0.40 (0.15–0.90)	0.40 (0.15–0.80)	0.641
Antidepressants	20 (5.5)	0.55 (0.15–1.53)	0.40 (0.15–0.80)	0.852
Antiepileptic drugs	82 (22.5)	0.55 (0.15–1.53)	0.40 (0.15–0.80)	0.902
Beta-blockers	40 (11.0)	0.28 (0.15–1.13)	0.40 (0.15–0.80)	0.256
Calcium channel blockers	5 (1.4)	0.15 (0.15–0.38)	0.40 (0.15–0.80)	0.743

Values are given as the medians and interquartile ranges or *n* (%). *p-value: from analysis of covariance between groups, with age and body mass index as covariates.

## Discussion

EM and CM present different characteristics and levels of some migraine biomarkers, but evidence of the CRP levels in EM and CM is still scarce. The present study found no significant difference in interictal CRP levels between participants with EM, CM, and healthy controls. Furthermore, CRP levels did not significantly vary in compliance with clinical characteristics, comorbidities, and preventive medication classes between participants with EM and CM. These findings reject the study hypothesis that the CRP levels are higher in participants with EM and CM than controls.

To date, 14 studies have reported CRP levels in individuals with migraine; however, the findings have been inconsistent (10–15, 18–25, 37). Although some studies showed elevated CRP levels in patients with migraine than in controls, other studies revealed no significant difference (Table 5). One possible explanation for the discrepancy in the association between CRP levels and migraine is the difference in the BMI distribution of the participants. BMI is an important factor affecting CRP levels (38), and we also found a significant linear association between CRP levels and BMI. In previous studies of adults classified as overweight or obese (≥25 kg/m^2^), the participants in the migraine group had higher CRP levels than those in the control group (11–15), except in one study from Belgium (10). In contrast, studies on adult participants with a mean BMI of normal weight or less (< 25 kg/m^2^) showed no significant discrepancy in CRP levels between the migraine and control groups (19–25), except in a Turkish study (18).

**Table 5:** Studies comparing C-reactive protein levels between adults with migraine and controls.

**Authors**	**Country**	**CRP levels during migraine**	**BMI in the control group**	**BMI in the migraine group**	**CRP in the control group (mg/L)**	**CRP in the migraine group (mg/L)**	**Comments**
Silva et al. (19)	Columbia	Non-significant	17.8 ± 2.5	MA: 18.3 ± 2.5 MO: 17.5 ± 2.6	0.46 ± 0.36	MA: 0.44 ± 0.26 MO: 0.66 ± 0.41	No significant difference in BMI
Vanmolkot et al. (10)	Belgium	Elevated	21.6 ± 2.8	22.6 ± 3.0	0.90 (0.36–1.79)*	1.42 (0.59–2.48)*	No significant difference in BMI
Kurt et al. (11)	The U.S.A.	Elevated	Not separately described	Not separately described	3.66 (mean)	3.97 (mean)	Women only
Guldiken et al. (18)	Turkey	Non-significant	25.1 ± 3.7	26.1 ± 5.6	0.053 ± 0.02	0.063 ± 0.024	No significant difference in BMI
Gudmundsson et al. (20)	Iceland	Non-significant	Middle aged: Men: 25.8 ± 3.5, Women: 25.2 ± 4.4; Young aged: Men: 23.1 ± 2.8, Women: 21.9 ± 2.8	Men, MA: 25.3 ±3.1, MO: 25.0 ± 4.6 Women, MA: 25. 0 ± 4.1, MO: 25.0 ± 4.6	Men: 0.83 (0.77–0.90)*, Women: 0.87 (0.79–0.97)*	Men: 0.79 (0.69–0.91)*, Women: 0.87 (0.75–0.99)*	5,906 middle-aged (55.0 ± 8.5 years) and 1,345 young (27.7 ± 5.5 years) adults
Tietjen et al. (12)	U.S.A.	Elevated	26.3 ± 0.6	28.7 ± 0.7	1.60 ± 0.6	3.96 ± 0.4	No significant difference in BMI
Hamed et al. (13)	Egypt	Elevated	25.03 ± 3.05	27.42 ± 4.5	0.72 ± 0.51	MO: 0.95 ± 0.47 MA: 0.92 ± 0.5	Higher BMI in the migraine group
Guldiken et al. (21)	Turkey	Non-significant	23.98 ± 3.18	26.54 ± 5.52	3.91 ± 3.89	4.28 ±4.01	Higher BMI in the migraine group
Yilmaz et al. (22)	Turkey	Non-significant	24.1 ± 3.6	24.9 ± 3.3	CRP >3.1, 10.0% (10/50)	CRP >3.1, 24.1% (15/62)	No significant difference in BMI
Theodoropoulos et al. (14)	The U.S.A.	Elevated	Not described	Not described	0.79 ± 0.31	2.08 ± 0.97	
Tietjen et al. (15)	U.S.A.	Elevated	25.87 ± 5.19	29.8 6 ± 7.49	Reference	Elevated CRP OR, 4.05 (1.56–10.51)^†^	Higher BMI in the migraine group
Güzel et al. (23)	Turkey	Non-significant	Not described	Not described	0.35 ± 0.16	MO: 0.12 ± 0.28 MA: 1.56 ± 0.72	
Rockett et al. (24)	Brazil	Non-significant	Non-obese: 22.2 ± 1.7 Obese: 34.7 ± 4.6	Non-obese: 22.8 ± 1.7 Obese: 33.3 ± 3.0	Non-obese: 4.0 (4.0–4.0)* Obese: 10.0 (4.0–12.4)*	Non-obese: 4.0 (4.0–4.0)* Obese: 4.0 (4.0–18.1)*	No significant difference in CRP levels among the four groups
Fava et al. (25)	Italy	Non-significant	22.5 ± 3.4	EM 23.1 ± 2.3 CM 22.6 ± 3.1	5.7 ± 1.4	EM: 5.6 ± 0.8 CM: 6.0 ± 3.2	No significant difference in BMI

*Median and interquartile range.^†^Odds ratios and 95% confidence interval. Values are given as the mean ± standard deviation unless otherwise noted. BMI, body mass index; CM, chronic migraine; CRP, C-reactive protein; EM, episodic migraine; MA, migraine with aura; MO, migraine without aura; OR, odds ratio.

These findings were similar to those concerning the relationship between CRP and depression. Depressive mood was highly related to elevated CRP levels in the obese or overweight population but not in the normal-weight population (39). Regarding migraine, a Brazilian study compared CRP levels between individuals with migraine and controls according to their BMI and found no significant discrepancy in CRP levels, both in the obese and normal-weight groups (24). However, the sample size was relatively small (*n* = 14 for the obese group and *n* = 15 for the normal-weight group); thus, these results might not accurately reflect the difference in CRP levels according to BMI.

The mean BMI in the present study was < 25 kg/m^2^, and no significant discrepancy in CRP levels was observed among the EM, CM, and control groups. Although CRP levels were not significantly different among individuals who were at least overweight (BMI ≥ 25 kg/m^2^) in the EM, CM, and control groups, CRP levels were elevated in the EM group when compared to the control group. However, only a small number of participants in the current study were at least overweight. Additional studies on CRP levels in individuals with migraine in various BMI groups will provide more information to identify the complex interaction between BMI, CRP, and migraine.

Another possible explanation for the conflicting findings is the difference in geographic distribution. Studies from Western Europe and North America showed significantly elevated CRP levels in participants with migraine than in controls (11, 12, 14, 15), whereas studies from other regions did not show significant differences (13, 18–25), except in a study from Egypt. These findings suggest a possible role of geographic distribution in the difference in CRP levels in individuals with migraine. Regarding depression, elevated CRP levels were associated with depressive symptoms in studies from Western Europe and North America but not in Asia and the Middle East (40–45). Considering that depression is a common comorbidity of migraine and shares pathogenic mechanisms with migraine, geographic distribution may be an explanation for the difference in the association between CRP levels and migraine noted among studies (32, 46).

However, as far as we are aware, no study thus far has compared CRP levels between individuals with migraine and controls in an Asian population. A Chinese study assessed CRP levels after pine needle moxibustion in individuals with migraine but did not compare them to non-migraine controls (47). The current study found that CRP levels were not related to the severity of anxiety and depression but were significantly associated with BMI. These findings are compatible with previous findings showing that CRP levels were not significantly associated with anxiety and depression (39, 48) but were associated with BMI in the normal-weight or underweight population (BMI < 25 kg/m^2^) (38). The similarity in findings between the present and previous studies suggests that the present study appropriately evaluated the association between CRP, anxiety, depression, BMI, and migraine.

To date, only one Italian study has evaluated CRP levels in individuals with CM, individuals with EM, and controls (25). The study found no significant discrepancy in CRP levels among the three groups. Similar results were found in the current study, and there were no significant discrepancies in CRP levels between the EM and CM groups. We also found that CRP levels were not statistically associated with clinical characteristics and comorbidities.

This study has some limitations. First, only female participants were enrolled to avoid the potential effects of sex differences on CRP levels. Sex differences in migraine and CRP have been reported. Women have a higher prevalence of migraine with more severe symptoms and disabilities than men (49). In addition, women generally have higher levels of CRP than men (50). Further, compared with men, women show a greater increase in CRP levels with increasing truncal fat (51). Therefore, our findings may not reflect CRP levels in men with EM and CM. Second, although age and BMI are important influencing factors of CRP levels (52, 53), the EM and CM groups were not matched for age and BMI, and there were significant differences between them. Nevertheless, age and BMI distributions were not statistically different between the EM and control groups or between the CM and control groups. Our analysis showed no statistical difference in CRP levels within the three groups, even with adjustments for age and BMI. However, our analysis of age and BMI between the EM and CM groups showed statistical significance. Third, although our study has a sufficient sample size in large categories, the subgroups have rather smaller sample sizes. Thus, the significance might be limited by the sample size. Specifically, the estimated sample powers between the EM and control groups and between the CM and control groups among participants who were at least overweight were 0.392 and 0.151, respectively. Nonetheless, we described these results to provide more information regarding CRP levels in participants with EM and CM.

## Conclusions

In conclusion, CRP levels are not statistically different between individuals with EM and individuals with CM and between individuals with migraine and healthy controls. Further, CRP levels do not significantly differ based on headache frequency, headache intensity, preventive treatment, MO, and FM. Therefore, CRP levels are not likely to be a biomarker for EM and CM. These findings clarify the significance of CRP levels in individuals with EM and CM in a Korean population.

## Data availability statement

The original contributions presented in the study are included in the article/supplementary material, further inquiries can be directed to the corresponding author/s.

## Ethics statement

The studies involving human participants were reviewed and approved by Institutional Review Board of Severance Hospital, Yonsei University (Approval No. 2018–2711-004). The patients/participants provided their written informed consent to participate in this study. Written informed consent was obtained from the individual(s) for the publication of any potentially identifiable images or data included in this article.

## Author contributions

CP conceptualized and designed the study, analyzed the data, and reviewed the manuscript. SL interpreted the data and drafted the manuscript. MC conceptualized and designed the study, collected and analyzed data, interpreted the data, and drafted the manuscript. All authors have read and approved the final manuscript.

## Funding

This research was supported by a grant from the Korea Health Technology R&D Project through the Korea Health Industry Development Institute (KHIDI), funded by the Ministry of Health & Welfare, Republic of Korea (Grant No.: HV22C0106) and a National Research Foundation of Korea (NRF) grant from the Korean government (MSIT) (2022R1A2C1091767).

## Conflict of interest

Author MC was a site investigator for a multicenter trial sponsored by Biohaven Pharmaceuticals, Allergan Korea, and the Ildong Pharmaceutical Company. He has received lecture honoraria from Eli Lilly and Company, Handok-Teva, and Ildong Pharmaceutical Company over the past 24 months. He received grants from Yonsei University College of Medicine (6-2021-0229) and the Korea Health Industry Development Institute (KHIDI) (HVC22CO106). Author CP was employed by Genuv Inc. The remaining author declares that the research was conducted in the absence of any commercial or financial relationships that could be construed as a potential conflict of interest.
