# Corrigendum: No change in interictal C-reactive protein levels in individuals with episodic and chronic migraine: A case-control study and literature review

**DOI:** 10.3389/fneur.2022.1123725

**Published:** 2023-01-05

**Authors:** Chae Gyu Park, Sue Hyun Lee, Min Kyung Chu

**Affiliations:** ^1^Heart-Immune-Brain Network Research Center, Department of Life Science, Ewha Womans University, Seoul, Republic of Korea; ^2^Laboratory of Immunology, Severance Biomedical Science Institute, Yonsei University College of Medicine, Seoul, Republic of Korea; ^3^Therapeutic Antibody Research Center, Genuv Inc., Seoul, Republic of Korea; ^4^Department of Neurology, Yonsei University College of Medicine, Seoul, Republic of Korea

**Keywords:** biomarker, C-reactive protein, inflammation, migraine, headache

In the published article, there was an error in the Funding statement. A National Research Foundation of Korea (NRF) grant was incorrectly omitted. The correct Funding statement appears below.

